# Protective Effects of Plant-Based Diets Against Colorectal Carcinogenesis via Modulation of Key Cellular and Molecular Mechanisms: A Comprehensive Review of Evidence

**DOI:** 10.3390/curroncol33040222

**Published:** 2026-04-17

**Authors:** Marina Kamel, Clarence Wong, Eduardo Grunvald, Andrea Galli, Sahar Iqbal, Arshdeep Rattol, Tanya Jackson, Sebastian Straube, Ellina Lytvyak

**Affiliations:** 1 School of Medicine, University of Galway, H91TK33 Galway, Ireland; m.kamel1@universityofgalway.ie; 2 Division of Gastroenterology, Department of Medicine, University of Alberta, Edmonton, AB T6G 2X8, Canada; ckw3@ualberta.ca; 3 Divisions of General Internal Medicine & Minimally Invasive Surgery, Department of Medicine, University of California San Diego, La Jolla, CA 92037, USA; egrunvald@health.ucsd.edu; 4 Gastroenterology Research Unit, Department of Experimental and Clinical Biomedical Sciences, University of Florence, 50121 Florence, Italy; 5 Faculty of Medicine, Memorial University of Newfoundland, St. John’s, NL A1B 3V6, Canada; 6 Faculty of Medicine and Dentistry, University of Alberta, Edmonton, AB T6G 2T4, Canada; 7 Division of Preventive Medicine, Department of Medicine, University of Alberta, Edmonton, AB T6G 2T4, Canada; 8 School of Public Health, University of Alberta, Edmonton, AB T6G 2G7, Canada

**Keywords:** colorectal cancer, carcinogenesis, plant-based diet, epigenetics, inflammation, dietary fibre, gut microbiota, short-chain fatty acids, phytochemicals

## Abstract

Colorectal cancer (CRC) remains one of the most prevalent malignancies worldwide and is the second largest contributor to both incidence and mortality, underscoring the urgent need for effective prevention strategies. This comprehensive review provides the most up-to-date evidence on the protective role of plant-based dietary patterns against CRC carcinogenesis, with particular emphasis on underlying cellular and molecular level mechanisms. Accumulating research demonstrates that plant-based foods, rich in dietary fibre, polyphenols, and multiple other bioactive compounds, promote gut microbial eubiosis, support immune regulation, and modulate adipose tissue homeostasis. These effects are accompanied by intestinal barrier integrity, enhanced production of short-chain fatty acids, and the induction of apoptosis in malignant cells. Moreover, plant-derived nutrients reduce the abundance of pro-inflammatory microbial taxa, decrease oxidative, nitrosative and carbonyl stress, and downregulate pro-inflammatory cytokines and signalling pathways, implicated in tumourigenesis. As a result, plant-based dietary patterns have high potential to reduce CRC risk through modulating the intricate interplay between epigenetics, inflammation, immune dysregulation, metabolic and hormonal disruptions, and gut microbiota, suggesting a highly promising, cost-effective and equitable strategy for CRC prevention.

## Simple Summary

Colorectal cancer is a highly prevalent malignancy that is significantly influenced by lifestyle factors, particularly dietary choices. This review synthesizes existing research indicating that plant-based diets offer protective benefits against colorectal cancer. Plant-based foods are abundant in fibre, antioxidants, and multiple other bioactive compounds that foster a healthier gut microbiome, mitigate inflammation, enhance immune function, and diminish genetic material damage. These mechanisms collectively decrease the likelihood of colorectal cancer development. Conversely, the consumption of red and processed meats, as well as poultry, is associated with cancer development via oxidative stress, inflammation, and the production of detrimental metabolites. Overall, adopting a plant-based diet represents a practical, affordable, equitable, and effective strategy to reduce colorectal cancer risk.

## 1. Introduction

### 1.1. Colorectal Cancer Epidemiology

Colorectal cancer (CRC) continues to represent a major global public health burden, despite being largely preventable and treatable if diagnosed early. In 2023, it contributed the third largest share of cancer incidence cases (8.9%), with 2.29 million people newly diagnosed [1]. It was also the second largest contributor to global cancer deaths (10.7%, 1.11 million) [1].

Between 1990 and 2023, the global number of incident CRC cases increased 2.5-fold, with a 2.1% rise in the age-standardized incidence rate, while the number of deaths almost doubled [1]. In 2023, CRC ranked as the 14th leading cause of death worldwide, rising from the 17th place in 1990, despite decreasing mortality rates [2]. Notably, the CRC burden is projected to rise dramatically, with the International Agency for Research on Cancer (IARC) estimating a 63% increase in new cases and a 73% rise in deaths by 2040, disproportionately affecting younger adults and populations in countries undergoing rapid economic transition [3]. From the perspective of disability burden, some regions, such as Central Europe and high-income Asia, had colorectal cancer among the top ten leading causes of disability-adjusted life-years (DALYs) [4].

Among adults between ages 40–64, CRC is the second leading cause of cancer incidence globally and the third leading cause of cancer mortality. In older adults above 65, CRC is one of the largest contributors to both cancer incidence and mortality, responsible for 52.7% of all cancer cases, 61.7% of all cancer deaths, and 40.9% of all cancer DALYs globally in 2023 [1,3]. Sex-wise, in males, over the last three decades, CRC had an 18.0% increase in 70q0, which is the measure quantifying the probability of dying before the age of 70 years, almost doubling the same estimate among females (9.4%) [2]. Moreover, in both sexes, in four global geographic super regions out of seven, along with the increased 70q0, the observed mean age at death from CRC was lower than expected [2]. Recent EPIC data shows that adherence to the 3V dietary pattern (low ultra-processed food intake, high plant-based diet (PBD) quality, and high food biodiversity) is associated with a significantly lower risk of CRC, with hazard ratios (HRs) around 0.84 compared with those with the lowest adherence [5].

The aim of this comprehensive review is to synthesize current epidemiological, clinical, and mechanistic evidence on the relationship between PBDs and CRC risk. Specifically, this review evaluates the biological pathways through which PBDs may influence colorectal carcinogenesis, integrates findings from observational studies, randomized controlled trials, and mechanistic research, and identifies gaps in the literature to inform future research and clinical guidance.

### 1.2. Non-Modifiable Colorectal Cancer Risk Factors

Several non-modifiable factors have been consistently associated with an increased risk of CRC. These include advancing age, which remains the most significant determinant of incidence, and also male sex, linked to higher susceptibility and reflected in a male-to-female ratio for CRC incidence of 1.4 [1,6]. A positive family history of CRC further elevates the risk, with a positive family history of CRC in a first-degree relative approximately doubling an individual’s lifetime risk [7]. Inherited high-penetrance genetic syndromes, such as Lynch syndrome and familial adenomatous polyposis [8], also elevate the risk. Hereditary syndromes account for 5–10% of all CRC cases, with Lynch syndrome being the most common; affected individuals have a lifetime CRC risk of 50–80% due to germline mutations in DNA mismatch-repair genes [9,10]. Familial adenomatous polyposis, caused by APC gene mutations, confers an almost 100% lifetime CRC risk without prophylactic colectomy [11]. Additionally, individuals with a personal history of colorectal neoplasia or inflammatory bowel disease (IBD) face a substantially higher risk [12]. Chronic inflammatory bowel diseases, including ulcerative colitis and Crohn’s disease, markedly elevate CRC risk, particularly with long disease duration, extensive colonic involvement, and persistent inflammation [13,14]. Epidemiological evidence further indicates that certain racial and ethnic groups, such as African Americans, Alaska Natives, and Eastern Europeans, exhibit differential susceptibility, likely reflecting a combination of genetic predisposition and potential environmental or socio-economic disparities [15]. More recently, rising CRC mortality rates have been reported among females of South Asian descent [2].

### 1.3. Modifiable Colorectal Cancer Risk Factors

To a large extent, the rising CRC burden has been attributed to global shifts toward Westernized lifestyles, leading to the increasing prevalence of obesity, diabetes, sedentary behaviour, and the abundance and affordability of foods with poor nutritional quality. Between 1990 and 2021, the rate of overweight and obesity doubled worldwide, and in many nations, it reached 80% [16,17]. Excess adiposity is a well-established risk factor for CRC, exhibiting itself via a systemic inflammatory state, increased estradiol, and hyperinsulinemia, leading to genome instability, reduced apoptosis, and altered macrophage function [18]. Meta-analyses demonstrate that overweight and obesity are associated with a 36% increased risk of developing CRC compared with individuals of normal weight [19]. Sedentary behaviour has also been associated with increased CRC risk; conversely, higher levels of regular physical activity are consistently linked to its risk reduction, with the most active individuals experiencing an approximate 20–30% lower risk compared to inactive ones [20]. Other modifiable risk factors include tobacco smoking and alcohol consumption. A 7% higher risk in CRC incidence was observed per 10 g/day increment of alcohol intake above zero, while smoking was associated with a 39% increased risk of left-sided CRC in males and a 20% increased risk of right-sided (proximal) CRC in females [21,22]. The Global Burden of Disease 2023 findings underscore a global epidemiological transition marked by declining communicable diseases and a rising burden of non-communicable diseases, including CRC, with nearly half of global disease burden being attributable to modifiable risks [20]. A recent pooled analysis of 1.8 million adults across nine prospective cohorts found that pescatarians had a modestly lower risk of CRC, whereas vegetarians showed no significant difference and vegans had a higher risk compared with meat eaters [23]. These findings suggest that simply excluding meat does not uniformly confer protection against CRC and that dietary quality and nutrient composition within PBDs may play a critical role. In particular, elevated risk observed among vegans may reflect potential deficiencies in protective nutrients or differences in food processing and substitution patterns. These findings reinforce CRC as a largely preventable malignancy, strongly linked to lifestyle.

Dietary patterns have emerged as an increasingly prominent focus in cancer epidemiology. PBDs, characterized by a high intake of fruits, vegetables, legumes, whole grains, nuts, and seeds while minimizing or eliminating animal-derived products, can be rich in fibre, antioxidants, and anti-inflammatory phytochemicals. These dietary patterns are increasingly popular due to their associations with reduced risk of cardiovascular disease (CVD), type 2 diabetes, obesity, and metabolic syndrome [24]. These metabolic improvements may contribute towards a reduced cancer risk, as metabolic dysregulation and chronic inflammation are shared risk factors between CVD, dyslipidemia, altered glycemic profile, and several types of cancer [25]. Other health benefits of PBDs include weight loss in the setting of obesity, normalization of serum cholesterol and low-density lipoprotein (LDL) levels, and improved blood pressure control found in multiple studies of vegetarians and vegans compared to individuals who opt for an omnivorous diet [26]. Furthermore, high dietary quality, such as adherence to plant-forward diet patterns, has been associated with improved overall survival and reduced mortality, particularly in breast cancer and CRC survivors [27]. Some studies have reported mixed results, suggesting that diet may need to be accompanied by other protective factors such as physical activity, healthy body weight, smoking cessation, and, perhaps, underlying genetic factors for maximum risk reduction [28].

A growing body of evidence points toward the potential of PBDs to act on multiple biological pathways involved in cancer initiation and progression [28]. PBDs have been associated with reduced levels of systemic inflammation, oxidative DNA damage, and insulin-like growth factors—key mediators of cancer pathogenesis [29]. Specifically, diets rich in plant foods have been shown to exert anti-proliferative, immunomodulatory, and anti-angiogenic effects that counteract carcinogenesis. However, the quality of plant-based foods matters; diets rich in whole grains, unsaturated fats, fruits, vegetables, and omega-3 fatty acids are most beneficial [30]. In CRC, in particular, dietary fibre promotes a healthier gut microbiota and stimulates the production of beneficial short-chain fatty acids like butyrate, which have been found to inhibit tumour cell growth and promote apoptosis [31]. Moreover, the IARC, a specialized agency of the World Health Organization, has classified processed meat as “carcinogenic to humans” and red meat as “probably carcinogenic to humans” [32]. Later, considering a growing body of evidence, the World Cancer Research Fund concluded that there is strong evidence that consuming red meat and consuming processed meat are causes of CRC [33]. Limiting red and processed meat intake, a key feature of PBDs, has been strongly associated with a reduced risk of CRC.

Epidemiological evidence consistently demonstrates that obesity is a major risk factor for CRC. Large prospective cohort studies show that individuals with obesity have approximately a 30–60% higher risk of developing CRC compared with those of normal weight, with the strongest associations observed for visceral adiposity [34]. Meta-analyses involving more than 6 million participants report a dose–response relationship, with CRC risk increasing by 5–10% for every five-unit increase in body mass index (BMI) [35]. Central obesity, measured by waist circumference or waist-to-hip ratio, appears to be an even stronger predictor of CRC risk rather than BMI alone, underscoring the role of metabolically active visceral fat in colorectal carcinogenesis [36].

## 2. Methodology

**Figure 1:**
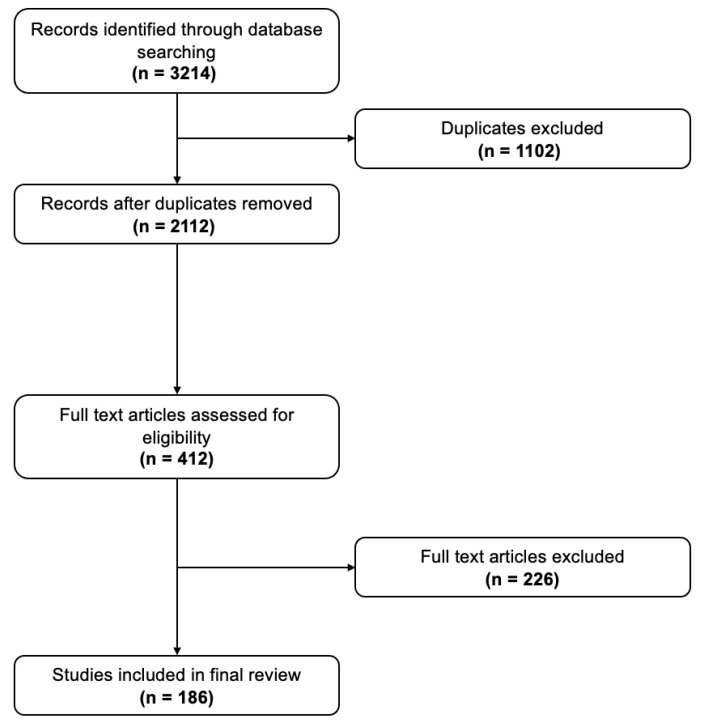
PRISMA flow diagram illustrating study selection process.

A comprehensive literature search was conducted to synthesize epidemiological, mechanistic, and clinical evidence on the relationship between PBD and CRC. For this review, “comprehensive” refers to the inclusion of systematic reviews, meta-analyses, randomized controlled trials when available, large prospective cohort studies, case–control studies, and mechanistic experimental research examining plant-derived dietary components and CRC-related outcomes. A structured search of PubMed, Scopus, and Google Scholar was performed for articles published up to February 2026 using a predefined search strategy combining terms such as “plant-based diet,” “vegetarian,” “vegan,” “colorectal cancer,” “CRC prevention,” “dietary fibre,” “phytochemicals,” “inflammation,” and “cancer risk.” The search yielded 3214 records; after removal of 1102 duplicates, 2112 titles and abstracts were screened. A total of 412 full-text articles were assessed for eligibility, and 186 studies met inclusion criteria, comprising 32 systemic reviews and meta-analyses, 78 prospective cohort studies, 12 randomized controlled trials, and 64 mechanistic or experimental studies (Figure 1). Studies were excluded if they did not report CRC-specific outcomes, did not examine plant-based dietary patterns or components, or lacked methodological clarity.

## 3. Meat and Poultry Consumption and Increased CRC Risk

### 3.1. Red and Processed Meat

Strong evidence from abundant prospective cohort and case–control studies identifies red and processed meat as a convincing cause of CRC, with a dose–response relationship [32,37,38,39]. Red meat is defined as any unprocessed mammalian muscle or organ meat, such as beef, pork, lamb, veal, goat, horse, mutton, and fresh organ meats [18,40]. Processed meat is defined as any meat or meat by-products that are preserved through fermentation, smoking, curing, salting, or the addition of other preservatives, as salt is a preservative [18].

The mechanistic basis for this association is well-established. Red and processed meat intake produces carcinogens through heme-iron-induced oxidative stress, formation of N-nitroso compounds, polycyclic aromatic amines and polycyclic aromatic hydrocarbons [40,41]. These compounds can induce DNA damage, inflammation, and carcinogenesis in colonic epithelial cells.

Beyond genetic mechanisms, red and processed meat may also promote colorectal carcinogenesis through lipid-mediated signalling pathways. Diets high in saturated fat elevate circulating long-chain fatty acids and arachidonic acid availability, enhancing cyclooxygenase-2 (COX-2)-mediated prostaglandin E2 production, which can activate peroxisome-proliferator-activated receptor delta (PPAR-δ), a downstream target of the adenomatous polyposis coli/βeta-catenin pathway implicated in tumourigenesis. This activation promotes proliferation, survival signalling, and inflammatory gene expression in colonic epithelial cells and may also further increase prostaglandin production, establishing a feed-forward interaction with COX-2-derived eicosanoids. A PBD likely reduces ligand-driven and inflammation-mediated activation of PPAR- δ, particularly in the colon [42,43,44].

Epidemiological studies provide compelling evidence supporting these mechanistic associations. A meta-analysis of prospective studies has shown that each additional 100 g/day of red meat consumption is associated with an approximately 14% increased risk of CRC, with the association appearing stronger for colon cancer than for rectal cancer [45]. Processed meat intake confers an even greater risk: a meta-analysis including 21 epidemiological studies found a 29% increased risk of colorectal adenomas for every 50 g/day increment in processed meat consumed [46].

In large prospective cohort studies, such as the Nurses’ Health Study and the Health Professionals Follow-Up Study with over 130,000 individuals included, it was demonstrated that consumption of processed meat was significantly associated with both overall CRC risk (HR = 1.15, 95% confidence interval (CI) 1.01–1.32, *p* = 0.03) and, specifically, distal colon cancer (HR = 1.36; 95%CI 1.09–1.69, *p* = 0.006) [47]. In a prospective European cohort including over 470,000 individuals over a mean follow-up of only 4.8 years, red meat intake was associated with a 25% higher risk of developing CRC (HR = 1.25, 95%CI 1.09–1.41, *p* = 0.001), while the consumption of processed meat conferred 55% higher risk per 100 g increase (HR = 1.55, 95%CI 1.19–2.02, *p* = 0.001) [48].

The National Institutes of Health Diet and Health study involving 500,000 participants aged 50–71 reported a 24% higher CRC risk in participants with the highest red meat intake, whereas the Adventist Health Study, involving 32,051 adults over the span of 6 years, found risk ratios of 1.85–1.90 for high total and red meat intake compared to non-consumers [49,50]. In a 4000-person Netherlands cohort study, heme iron intake was associated with a 70% higher risk of CRC [51]. Similarly, Japanese cohorts have identified sex-specific associations, with high red meat intake in females and high total meat consumption among males linked to an increased risk of CRC [52].

Overall, evidence from meta-analyses, large population-based prospective cohorts, and case–control and mechanistic studies strongly supports the IARC classification of processed and red meat as carcinogenic agents, reinforcing the recommendation to limit red and processed meat intake as part of a CRC prevention strategy [47].

### 3.2. Poultry

Poultry encompasses all types of birds, such as chickens, turkeys, geese, ducks, quail and pheasants [53]. Compared with red meat, poultry is typically lower in fat content and contains a higher proportion of white muscle fibres. Even though poultry is leaner than red meat, it is still a source of heme iron and saturated fat, which are associated with pro-oxidative carcinogenic effects. Moreover, grilling, frying, or broiling poultry, or any exposure to high temperatures, forms heterocyclic amines and mutagenic compounds, the same as those produced when cooking red meat this way, thereby exhibiting the same tumour-promoting effects. Advanced glycation allows these compounds to induce oxidative stress and inflammation, which are implicated in CRC pathogenesis [33]. Processed poultry can include nitrates, nitrites, and high sodium levels, which are implicated in CRC development [33].

More recently, a body of evidence linking poultry to the increased CRC risk has been growing. A large 4800-individual prospective study conducted in Italy and Spain reported that males consuming more than 300 g of poultry per week had a 2.61-fold higher risk of mortality from gastrointestinal cancers compared with those consuming less than 100 g per week (HR-2.61, 95%CI 1.31–5.19, *p* < 0.05) [53]. A prospective cohort study from Denmark with 6282 participants showed that higher poultry intake was linked to a 62% increased overall risk of CRC (HR = 1.62, 95%CI 1.13–2.31, *p* < 0.05) [54].

## 4. Cellular and Molecular Mechanisms of PBD Impact on CRC Pathogenesis

### 4.1. Epigenetics

**Figure 2:**
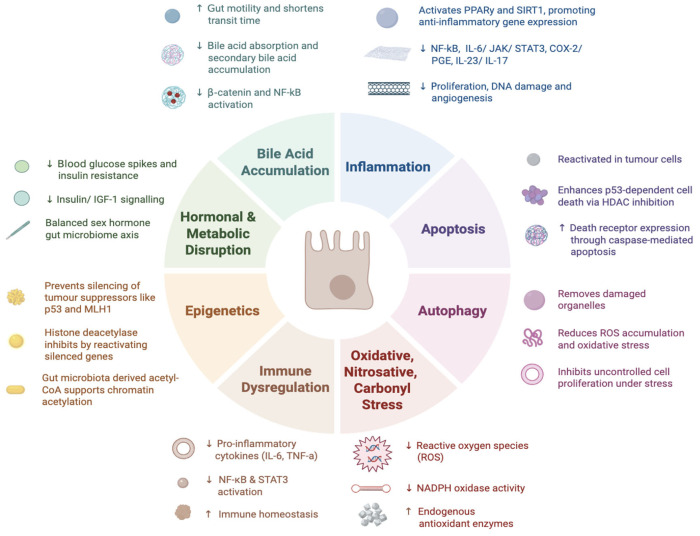
Impact of plant-based diet on cellular and molecular pathways in colorectal carcinogenesis. Created in BioRender by M. Kamel (2025). https://app.biorender.com/illustrations/693c710d94088d47f877e032?slideId=1e679e93-54a3-4bcd-a915-aa8b9512fae3 (accessed on 7 April 2026).

Epigenetics refers to heritable changes in gene expression that occur without alterations to the underlying DNA sequence [aberrant DNA hypermethylation can turn off tumour suppressor genes 55 (Figure 2). The primary epigenetic mechanisms include DNA methylation and histone modification. In CRC,thereby promoting carcinogenesis [55]. For example, hypermethylation-mediated inactivation of genes such as p53 and MHL1 contributes to genomic instability and tumour progression. Folate and vitamin B6, which are abundant in plant-based foods such as chickpeas, potatoes, and whole grains, play essential roles in one-carbon metabolism and DNA methylation processes [55]. Adequate intake of these micronutrients supports appropriate methylation patterns and may help prevent the epigenetic silencing of tumour suppressor genes [55]. At the chromatin level, gene expression is regulated by the dynamic balance between histone acetylation and deacetylation [56]. This process is mediated by histone acetyltransferases and histone deacetylases. The main source of acetyl for these reactions is acetyl-CoA, produced by the gut microbiota. When a PBD re-establishes a healthy gut microbiome balance, it indirectly shapes the epigenome, making cells more resistant to tumour initiation and lowering CRC risk via increasing acetyl CoA availability via acetate. Sulphoraphane and curcumin, found in broccoli and turmeric, can modify histones, leading to the inhibition of histone deacetylases [57,58]. This can then reactivate silenced tumour suppressor genes and slow cell proliferation to allow apoptosis to occur in precancerous colon cells.

### 4.2. Inflammation

Chronic inflammation is a major contributor to the development of CRC development, promoting cellular proliferation, angiogenesis, genomic instability and tumour progression. Diet plays a central role in modulating inflammatory status. Diets high in red and processed meats, saturated fats, and refined carbohydrates are known to induce a pro-inflammatory state [59]. In contrast, PBDs, rich in anti-inflammatory components such as polyphenols, omega-3 fatty acids, antioxidants, vitamins, and dietary fibre, are associated with reduced systemic inflammatory markers, including C-reactive protein, interleukin-6 (IL-6), and tumour necrosis factor-alpha (TNF-α).

Persistent colonic inflammation creates sustained oxidative stress, leading to DNA damage by oxidizing nucleotide bases (particularly guanine) and causing DNA strand breaks, which can lead to a phenomenon known as “field cancerization” [60]. This process allows dysplastic changes to arise in multiple areas of the colon, increasing the likelihood of tumour development [60]. This mechanism is particularly evident in patients with IBD, in whom chronic immune activation accelerates the inflammation–dysplasia–cancer sequence [61].

At the molecular level, chronic inflammation activates several oncogenic signalling pathways, most notably nuclear factor kappa B (NF-κB). NF-κB signalling induces transcription of pro-inflammatory cytokines such as IL-6, IL-1β, and TNF-α, as well as reactive oxygen species, all of which contribute to DNA damage and tumour promotion [60,61,62]. IL-6 further drives tumourigenesis through activation of the JAK/STAT3 pathway, enhancing cell cycle progression and inhibiting apoptosis [60,62]. These inflammatory cascades are self-reinforcing, creating a sustained pro-tumourigenic environment [62].

A critical regulatory mechanism in inflammation-driven carcinogenesis involves peroxisome-proliferator-activated receptor gamma (PPAR-γ). This nuclear receptor suppresses NF-κB signalling, reduces inflammatory mediator production, and promotes apoptosis of damaged epithelial cells [59]. Activation of PPAR-γ by dietary ligands, including omega-3 fatty acids, polyphenols, and metabolites from probiotic fermentation, can modulate gut inflammation and interrupt the inflammation–dysplasia–carcinoma sequence. Diets rich in these bioactive compounds may reduce CRC risk by enhancing PPAR-γ activity, maintaining gut epithelial integrity, and attenuating chronic inflammatory responses [63]. In healthy individuals, adherence to a vegetarian diet is associated with significantly fewer single-strand DNA breaks in exfoliated colorectal mucosal cells compared with consumption of a meat-rich diet [59].

The interactions between chronic inflammation, diet, and microbiota further shape colorectal tumourigenesis. The tumour microenvironment, composed of immune cells, stromal cells, and the gut microbiome, plays a dynamic role in either promoting or restraining tumour development. Persistent inflammatory signalling, particularly through IL-6/JAK/STAT3 activation, enhances epithelial proliferation and suppresses apoptosis. A pro-inflammatory diet promotes microbial dysbiosis, whereas anti-inflammatory dietary patterns support microbial diversity, reinforce epithelial barrier integrity, and modulate immune responses toward homeostasis [62].

Inflammation is also tightly linked to epigenetic regulation in CRC. Sirtuin-1 (SIRT1), an NAD+-dependent deacetylase, suppresses inflammatory signalling through inhibition of NF-κB activity. Experimental studies demonstrate that SIRT1 can limit intestinal tumourigenesis and colon cancer growth by restraining NF-κB-mediated transcription [64]. However, SIRT1 is overexpressed in specific CRC subtypes and has been associated with microsatellite instability and the CpG island methylator phenotype [65]. Cancer-specific functions of SIRT1 may also promote epithelial cancer growth and survival [66]. These findings suggest a dual role for SIRT1 in CRC: protective through modulation of inflammatory signalling, yet potentially pro-tumourigenic depending on molecular context.

The acetylation status of non-histone proteins also contributes to CRC development. The tumour suppressor p53 requires acetylation for full activity, and histone deacetylase 1 can directly deacetylate p53, reducing its ability to induce cell cycle arrest and apoptosis [67]. This loss of p53 function promotes survival of genetically damaged cells and supports colorectal carcinogenesis.

Overall, chronic inflammation contributes to DNA damage, activation of early oncogenic pathways, epigenetic alterations, and immune dysregulation, ultimately favouring tumour survival and growth. Key inflammatory pathways related to the early phases of CRC development include IL-23, which induces IL-17, and cyclooxygenase-2, which produces prostaglandins (PGEs) that drive inflammatory responses [68]. NF-κB, activated by TNF-α, IL-1, and reactive oxygen species (ROS), plays a central role in regulating inflammation [68]. Polyphenols found in fruits, vegetables, tea, and whole grains can activate SIRT1 expression and enhance deacetylase activity, thereby suppressing NF-κB signalling and reducing inflammatory cytokines such as TNF-α and IL-6 [64,66].

### 4.3. Immune Dysregulation

Immune dysregulation occurs when the immune system becomes overly reactive or underreactive, driving chronic inflammation and the progression of CRC [69]. Disruption of normal gut–immune interactions fosters a pro-tumourigenic microenvironment, characterized by aberrant cytokine signalling, impaired epithelial immune tolerance, and microbial imbalance [60,61,69]. A PBD may help restore immune homeostasis by modulating several interconnected gut immune pathways.

A central mechanism linking diet to immune regulation involves fermentation of dietary fibre by the gut microbiota into short-chain fatty acids (SCFAs), such as butyrate, acetate, and propionate. SCFAs exert immunoregulatory effects by promoting regulatory T-cell (Treg) differentiation, inhibiting pro-inflammatory pathways like NF-κB and STAT3, and maintaining mucosal immune tolerance [70,71]. They also enhance epithelial barrier integrity by enhancing tight junction protein expression, thereby limiting the translocation of microbial antigens that otherwise drive immune activation. In CRC, depletion of SCFA-producing bacteria reduces Treg development and facilitates sustained inflammatory signalling [70]. Following their production in the colon, SCFAs are rapidly absorbed by colonocytes and enter systemic circulation, enabling broader immunomodulatory effects beyond the intestinal environment [71].

Beyond SCFAs, immune dysregulation in CRC is further shaped by alterations in bile acid and tryptophan metabolism. Dysbiosis alters bile acid composition, decreasing levels of anti-inflammatory secondary bile acids while increasing cytotoxic bile acids, which promote epithelial stress and inflammation [70]. Simultaneously, in CRC and dysbiosis, the mechanism by which tryptophan activates aryl hydrocarbon receptors (AhRs) is impaired, leading to reduced AhR agonists and increased inflammation [70].

Cytokines play a central role in modulating immune responses and inflammation in CRC. Elevated levels of pro-inflammatory cytokines, including TNF-α, IL-6, and IL-17, contribute to tumour initiation, immune evasion, and disease progression [72]. IL-6-mediated activation of the JAK/STAT3 pathway promotes epithelial proliferation and inhibits apoptosis, thereby supporting tumourigenic processes [72,73]. Chronic activation of NF-kB by cytokines such as TNF-α further perpetuates a pro-inflammatory tumour microenvironment conducive to cancer growth [73].

PBDs have been shown to attenuate this immune dysregulation by reducing systemic and local inflammatory cytokine levels [74]. Polyphenols, as well as dietary fibres, fermented into SCFAs, like butyrate, downregulate the production of TNF-α, IL-6, and IL-1β while enhancing the expression of anti-inflammatory cytokines like IL-10. In addition, plant-based nutrients modulate chemokine expression and influence the recruitment and activity of myeloid-derived suppressor cells and tumour-associated macrophages, key mediators of immune suppression within the tumour microenvironment [70,74].

### 4.4. Oxidative, Nitrosative, and Carbonyl Stress

Oxidative stress arises from an imbalance between the production of ROS and the body’s antioxidant defense systems [75]. In the colon, sustained oxidative stress damages DNA, lipids, and proteins, contributing to tumourigenesis. Key sources of ROS in colorectal tissues include inflammatory cells such as macrophages, neutrophils, and NADPH oxidases (NOXs), which are often upregulated in chronic inflammation and tumour microenvironments [76]. NOX-mediated ROS production promotes CRC progression by activating pro-inflammatory signalling pathways.

Oxidative stress plays a pivotal role in CRC development, as mentioned above, through dysregulated ROS production by NADPH oxidase (NOX) enzymes, particularly NOX1, which is highly expressed in colonic epithelial cells [77]. NOX1-derived ROS drives signalling pathways involved in tumour growth and epithelial proliferation through mechanisms dependent on the activation of Rac1 and the recruitment of subunits NOXO1 and NOXA1 [77]. Beyond NOX1, other ROS-generating systems, including xanthine oxidase, nitric oxide synthase, and cytochrome P450 enzymes further amplify oxidative imbalance in the colonic microenvironment [78].

Nitrosative stress is an imbalance driven by the excessive production of reactive nitrogen species. Elevated biomarkers include nitric oxide, S-nitrosothiols, peroxynitrite, and nitrotyrosine [79]. Increased nitrosative stress in CRC is accompanied by increased levels of protein and lipid oxidation, and elevated pro- and anti-inflammatory cytokines, such as IL-1a, IL-6, and IL-10, were observed [79].

Reactive carbonyl species further contribute to cellular damage through the formation of advanced lipoxidation end products that bind to DNA and proteins, altering their function [80]. Carbonyl compounds can further damage cellular proteins and membranes, exacerbate oxidative stress, and create a positive feedback loop mechanism. Individuals with a detoxifying enzyme deficiency, such as aldo-keto reductase 1B10, are more vulnerable to the effects of carbonyl stress, as these enzymes usually help neutralize reactive carbonyl species [80]. Together, both carbonyl stress and nitrosative stress act together, contributing to mutations, genomic instability, and epigenetic dysregulation that drive colorectal carcinogenesis.

A PBD offers a protective counterbalance to this dysregulation by supplying a broad range of antioxidants that interrupt oxidative chain reactions and enhance endogenous cellular defense systems. These compounds also suppress ROS-producing enzymes and modulate redox-sensitive transcription factors involved in tumour progression [81]. Antioxidants, such as vitamins C and E, flavonoids, and polyphenols, neutralize ROS, enhance endogenous antioxidant enzyme activity, and inhibit NOX enzymes, thereby reducing oxidative DNA damage in colorectal epithelial cells [82].

### 4.5. Autophagy

Autophagy is generally considered a tumour-suppressive mechanism, serving as an adaptive cellular response to numerous extracellular and intracellular stresses, including nutrient and growth factor deprivation and hypoxia [83]. As the principal intracellular catabolic pathway, autophagy is uniquely capable of degrading damaged organelles—particularly mitochondria generating excessive reactive oxygen species (ROS)—thereby limiting oxidative stress and preserving genomic integrity. In the gastrointestinal tract, defective autophagy has been linked to immune dysregulation and chronic inflammation. Impaired autophagic flux promotes the accumulation of damaged mitochondria and intracellular stress signals, fostering pro-inflammatory microenvironments that favour tumour initiation and progression [83]. Under physiological stress conditions, disruption of nutrient- and growth-factor-dependent signalling pathways activates autophagy while concurrently suppressing anabolic processes, cell growth, and proliferation. This coordinated response supports cellular adaptaion and survival; however, when dysregulated, it may contribute to inflammation-associated carcinogenesis [84].

### 4.6. Apoptosis

PBD components such as phenols, flavonoids, and alkaloids may help correct abnormal post-transformational modifications linked to CRC and stimulate apoptosis in tumour cells [85]. In CRC, several proteins show abnormal post-transformational methylation, which reduces the function of growth-suppressive proteins, increases expression of anti-apoptotic regulators, such as BCL2, and impairs p53- mediated pro-apoptotic signalling [85,86]. This dysregulation allows tumour cells to evade programmed cell death [86].

A PBD can help reduce the risk of dysregulated apoptosis, thereby reducing the risk of CRC. Dietary fibre, particularly butyrate, acts as a histone deacetylase inhibitor, enhancing the p53-mediated apoptosis pathway and upregulating death receptor expression [87,88]. Similarly, in cell studies using bioactive compounds from fingerroot, elevated ROS led to apoptotic death in HT-29 and HCT116 CRC cells via caspase activation [89]. Collectively, these plant-derived compounds demonstrate the potential to restore apoptotic mechanisms.

### 4.7. Hormonal, Metabolic, and Adipose Tissue Disruptions

Hormonal disruption refers to an imbalance in the level of hormones in the body, which can affect growth and metabolism [90]. Metabolic disruption impairs regulation of processes such as blood glucose control and energy production, creating an environment that increases CRC risk. High insulin levels and elevated IGF-1 promote cell proliferation and inhibit apoptosis [90]. Hormonal imbalance may also alter gene expression through disrupted methylation processes. In addition, disruption of sex hormones, such as oestrogen, influences the gut microbiome by supporting growth of SCFA-producing bacteria [91]. β-glucuronidase activity further modulates this axis by deconjugating oestrogens and androgens, linking microbiome alterations to CRC progression.

Adipose tissue functions as an active endocrine organ that contributes to both metabolic and inflammatory processes [92,93]. Excess adiposity promotes chronic, low-grade inflammation through upregulation of pro-inflammatory cytokines, such as IL-6, and activation of oncogenic pathways, such as STAT, IL-6, MAPK, and PI3K [92]. This inflammatory milieu contributes to CRC initiation and progression while impairing immune surveillance through altered T-cell and B-cell signalling. Dysregulated adipokine secretion further contributes to tumourigenesis, with elevated leptin promoting proliferation and angiogenesis and reduced adiponectin removing anti-proliferative and pro-apoptotic effects [92,93].

Visceral adipose tissue (VAT) plays a central role in metabolic dysfunction, exhibiting increased lipolysis and fatty acid release compared to subcutaneous adipose tissue, thereby increasing the risk of resistance [94]. This contributes to hyperinsulinemia, elevated insulin-like growth factor-1 (IGF-1) levels, and increased free fatty acids, all of which exacerbate hyperglycemia and promote CRC development [94,95]. These metabolic changes activate key oncogenic pathways, including PI3K/Akt/mTOR and MAPK pathways, and support the Warburg phenotype, in which tumour cells preferentially rely on aerobic glycolysis for rapid growth [92,96]. Reduced circulating IGFBP-1 and IGFBP-2 further amplify IGF-1 signalling and tumour progression [96,97].

Dietary fat composition influences adipose tissue behaviour and CRC risk. Saturated fatty acids promote lipid accumulation in VAT, whereas monounsaturated fats favour storage in SAT and reduce visceral adiposity [94,98]. Additionally, adipose tissue reflects long-term dietary fatty acid intake and serves as a biomarker of diet–cancer interactions [99]. Higher levels of omega-3 fatty acids, particularly alpha-linolenic acid (ALA) found in plants, have been associated with a lower risk of CRC, whereas higher omega-6 levels and their enzymatic activity may be linked to an increased CRC risk [99].

Dietary patterns such as PBDs may mitigate these effects by improving insulin sensitivity, reducing visceral adiposity, and modulating inflammatory and metabolic pathways [93,94,100].

Metastasis is the leading cause of mortality in CRC [101]. Thyroid hormone receptor-interacting protein 6 (TRIP6) contributes to cancer progression by promoting cell migration, invasion, and metabolic reprogramming. Through activation of the Akt signalling pathway via interaction with PARD3, TRIP6 enhances glycolytic activity and suppresses tumour suppressor pathways, facilitating tumour growth and metastatic potential [101].

### 4.8. Bile Acid Accumulation

Accumulation of secondary bile acids, such as deoxycholic acid and lithocholic acid, leads to activation of β-catenin and NF-kB pathways, promoting proliferation and inflammation because BA receptors, such as FXR and TGR5, act as mediators of inflammation and carcinogenesis. Microbiome studies show that bai genes, SBA-producing genes, are enriched in CRC patients [102]. Physiologically, primary bile acids are conjugated with glycine or taurine in the liver and stored in the gallbladder. Following ingestion of a fat-rich meal, cholecystokinin stimulates gallbladder contraction, leading to bile acid release into the small intestine to facilitate lipid digestion. Increased dietary fat intake enhances bile acid secretion, thereby increasing substrate availability for microbial conversion into secondary bile acids in the colon.

In contrast, PBDs, characterized by high fibre and low fat contents, decreases bile acid secretion [102]. This prevents secondary bile acids from accumulating in the colon and damaging epithelial cells. A PBD allows the gallbladder and intestine to contract faster and increase gut motility, thereby shortening transit time and allowing bile acids to be cleared faster. Fibre in plants also binds bile acids, reducing their absorption.

### 4.9. Butyrate Production

Dietary fibre, abundant in PBDs, is one of the most studied protective factors against CRC [103]. Fibre undergoes fermentation by gut microbiota in the colon, leading to the production of short-chain fatty acids, particularly butyrate. This molecule plays a vital role in maintaining colonic health by serving as the primary energy source for colonocytes, regulating cell proliferation and apoptosis, and reducing inflammation and oxidative DNA damage. The protective role of fibre has been recognized by the WHO, and modern industrialized diets, often low in fibre, are associated with gut microbiota dysbiosis and increased CRC risk [104]. In contrast, rural African populations consuming >50 g/day of dietary fibre exhibit remarkably low CRC incidence, supporting a potential protective effect of high fibre intake; however, these observations may be confounded by a shorter life expectancy, as age-standardized incidence data are limited [104].

### 4.10. Gut Microbiota and Its Role

In CRC, the gut microbiota undergoes dysbiosis, which is an imbalance in the diversity and function of the microbes in the gut. This results in the gut microbiome having beneficial and more harmful bacteria, such as elevated levels of *Fusobacterium nucleatum*, which promotes inflammation [105].

The gut barrier is composed of the mucous layer, composed of mucins secreted by goblet cells that physically separate microbes from epithelial cells; the epithelial layer, sealed by tight junction proteins; and the immune layer, composed of dendritic cells, macrophages, T-cells and plasma cells [106]. Bacterial metabolites can cross the barrier in CRC. SCFAs cross by passive diffusion or via monocarboxylate transporters such as MCT1 or SMCT1, while hydrogen sulphide can diffuse across cell membranes [105,106].

A compromised gut barrier increases intestinal permeability, allowing microbial products to translocate into the circulation and trigger systemic inflammation, a key driver of tumourigenesis. PBDs promote the growth of beneficial gut microbiota in contrast to Western dietary patterns high in fat and animal proteins; PBDs enhance microbial diversity and boost SCFA production. SCFAs then suppress inflammation and induce apoptosis in the CRC cells [106,107].

The gut microbiota includes bacteria, viruses, fungi, and archaea. Studies show that microbial dysbiosis is a hallmark of CRC, with increased abundance of pro-tumourigenic bacteria such as *Fusobacterium nucleatum*, *Escherichia coli*, *Bacteroides fragilis*, and *Peptostreptococcus anaerobius* [107]. These contribute to genotoxicity, leading to DNA damage, and pathogens modulating pattern recognition receptor (PRR) signalling, enhancing chronic inflammation [107]. Early-stage adenomas show distinct shifts in the gut microbiota, including enrichment of *Actinomyces odontolyticus* and *Atopobium parvulum*, highlighting their potential use as biomarkers for CRC screening [108]. Non-bacterial organisms engage in inter-kingdom interactions, amplifying microbial pathogenicity. For instance, co-enrichment of fungi like *A. rambelli* and pathogenic bacteria such as *F. nucleatum* enhances CRC prediction over bacterial signals alone [108].

Enrichment of *Aspergillus rambelli* and *Malassexia* fungi and increased *Basidiomycota*-to-*Ascomycota* ratios have been observed in CRC and adenomas. Increased halophilic archaea and decreased methanogenic archaea have been associated with CRC [108]. In fewer studies, changes in the microbiome also resulted in higher levels of *Malassezia* fungi. *F. nucleatum* and *Solobacterium moorei* bacteria increased in abundance in the early-to-late stages of carcinogenesis, while *Atopobium parvulum* and *Actinomyces odontolyticus* bacteria levels increased only in the early stage of carcinogenesis [108].

PRRs serve as critical interfaces between the gut microbiota and the host immune system. Upon recognizing microbial antigens, PRRs activate a cascade of signalling molecules [109]. Specifically, *F. nucleatum* can activate TLR4 signalling to promote tumour development, while *Peptostreptococcus anaerobius* can promote carcinogenesis via activation of the TLR4 pathway. Bile acids represent another class of metabolites closely related to diet and the gut microbiome. They are steroid acids originally synthesized by the liver and metabolized to secondary acids in the intestines by bacteria. Secondary bile acids, such as deoxycholic acid, are produced from primary bile acids by intestinal bacteria and are influenced by dietary patterns, with high-fat diets increasing their levels. These metabolites can cross the epithelial barrier via passive diffusion and act as pro-inflammatory molecules, contributing to CRC risk [104,105,106].

## 5. Impact of Specific Plant-Based Diet Components on Colorectal Carcinogenesis

### 5.1. Antioxidants

In addition to macronutrients, such as carbohydrates, protein, and fat, PBDs provide polyphenols that are metabolized by the gut microbiota into bioactive compounds with anti-inflammatory and chemopreventive properties. Compounds found in cruciferous vegetables, berries, garlic, and herbs such as fenugreek exhibit antioxidant, anti-inflammatory, and anti-proliferative properties. These compounds act on molecular pathways related to carcinogenesis, such as oxidative stress, inflammation, and DNA repair. However, some evidence in this domain stems from in vitro studies using high-dose extracts not typically consumed in diets and provides mainly mechanistic insights [110].

A PBD is inherently rich in exogenous antioxidants, which neutralize free radicals, thereby neutralizing oxidative damage [111]. Examples include carotenoids and flavonoids that modulate inflammatory signalling and other key cancer-related pathways, such as NF-kB suppression [111]. Natural phytochemicals, particularly polyphenols, can inhibit MRP2 expression and its promoter by inhibiting NF-kB-Nrf2 signalling in HCT116/L-OHP and HCT-8/VCR cell lines. This interaction with the gut microbiota promotes beneficial taxa and reduces pro-tumourigenic activity, further contributing to reduced CRC risk.

### 5.2. Vitamins

Micronutrients, including vitamins, minerals and trace elements, are essential constituents of the diet and play important roles in maintaining cellular homeostasis and genome integrity [33]. Vitamins provide antioxidant defense, such as vitamins C and E, which donate their electrons to free radicals to neutralize reactive oxygen species (ROS), thereby reducing oxidative DNA damage [112,113]. Vitamin E also protects cell membranes from lipid peroxidation [113]. Cereal, which is part of a PBD, contains variable amounts of vitamins B and E, leading to a lower CRC risk [33,114]. Vitamin B9 (folate), found in legumes and grains, donates methyl groups, which maintain DNA methylation balance [115]. Proper DNA methylation helps maintain genomic stability by preventing the silencing of tumour suppressor genes and inappropriate activation of oncogenesis [115,116]. Vitamin D has also been implicated in CRC prevention and can modulate immune signalling, in part by suppressing pro-inflammatory cytokines [33,117]. If all these factors are regulated via vitamins in a balanced PBD, CRC risk is reduced [33,114].

### 5.3. Minerals

Vegetables and fruits are sources of many minerals. Selenium is a cofactor for glutathione peroxidase, which neutralizes ROD and reduces DNA damage. Selenium also has anti-inflammatory effects by activating NF-kB [118]. A prospective study found that higher dietary selenium intake was associated with improved CRC-specific survival, suggesting a protective role for selenium in influencing outcomes after diagnosis [118]. If the body is deficient in selenium, protective selenoproteins are not expressed, compromising antioxidant defenses [33].

Calcium is found mostly in milk and in leafy greens, such as broccoli. Dietary calcium reduced the risk of colorectal adenomas, which are known precursors to CRC, by 21% [119]. Calcium binds secondary bile acids, limiting their ability to damage the colon epithelia. Calcium has a growth-inhibiting action on tumour gastrointestinal cells and limits chronic inflammation by playing a role in tight junction integrity. Adequate calcium also helps regulate apoptosis in colon cells.

Phosphorus is essential for ATP, DNA, and phospholipid synthesis. Therefore, high dietary phosphorus can enhance Akt signalling and cell proliferation, increase ROS production, and support tumour growth [120]. However, in PBDs, phosphorus is largely found as phytate, which is poorly absorbed. This results in a lower effective phosphorus burden and allows plant-based diets to still be protective against CRC [118].

### 5.4. Dietary Fibre

Dietary fibre is a non-digestible component of plant-based foods that plays a central role in maintaining colon health through modulating gut microbiota, producing SCFAs, and reducing sustained immune activation. Epidemiological and mechanistic evidence consistently demonstrate that higher dietary fibre intake is associated with reduced proinflammatory cytokines like IL-6 and TNF-α and therefore a reduced risk of CRC [121].

Dietary fibre also plays a crucial role in preserving gut barrier integrity. In a healthy colon, a mucus layer and tight junctions prevent translocation of pathogenic microbial components, while SCFAs are selectively transported across epithelial cells. In CRC, barrier disruption allows microbial products such as lipopolysaccharides (LPSs), flagellin, and peptidoglycan to cross the epithelium [122]. Lipopolysaccharides from gram-negative bacteria leak through damaged tight junctions and trigger toll-like receptor (TLR) 4, activating NF-kB, and thereby increasing IL-6 and TNF-a, causing chronic inflammation [122]. Bacterial flagellin crosses via leaky tight junctions or transcytosis and binds TLR5 on epithelial cells, causing NF-κB-dependent inflammatory signalling [123]. Peptidoglycan fragments are internalized and sensed via endocytosis by NOD1/NOD2 receptors in epithelial cells to activate innate immune responses and promote inflammation [124]. Within the gut, enterotoxins disrupt epithelial tight junctions, allowing for further leakage and degradation of proteins such as E-cadherin, promoting inflammation and cell proliferation [125]. In contrast, by promoting SCFA production, dietary fibre helps maintain epithelial tight junctions and limits exposure to pro-inflammatory microbial antigens.

Higher soluble fibre intake is associated with lower CRC risk, with an effect that can be influenced by genetic background. A pooled analysis of 21 large prospective studies found that individuals in the highest fibre intake group had a significantly lower risk of CRC than those in the lowest group [126]. Dose–response analyses further support this relationship, with a hazard ratio of 0.90 per 5 g/1000 kcal increment, meaning a lower risk of CRC from higher fibre intake [126]. Similarly, the European prospective investigation into cancer and nutrition study demonstrated a 40% reduction in CRC risk with the highest quantile of fibre intake [48], while increasing the intake of whole grains by 90 g/day resulted in a 20% lower CRC risk [127].

Both soluble and insoluble fibre consumption appear to be protective against CRC, with a clinically significant comparable reduction in CRC risk [128]. Insoluble fibre, predominantly found in cereal grains, increases stool bulk and shortens colonic transit time, thereby reducing the duration of mucosal exposure to carcinogenic compounds. Soluble fibre, which is abundant in fruits and vegetables, is more readily fermented by gut microbiota to produce SCFAs with anti-inflammatory and anti-carcinogenic properties in the colon [70]. Soluble fibres dissolve in water and gastrointestinal fluid, while insoluble fibres do not dissolve [126]. This causes insoluble fibre to undergo slower fermentation and prevents constipation, allowing for a speedier gut movement. Meta-analyses indicate that total, soluble, and insoluble fibre intakes are similarly associated with reduced CRC risk: total fibre (effect size (ES) = 0.75, 95%CI 0.66–0.86), soluble fibre (ES = 0.78, 95%CI 0.66–0.92), and insoluble fibre (ES = 0.77, 95%CI 0.67–0.88) [128].

A study showed soluble fibre intake interacts with a specific genetic variant, rs4730274, near the SLC26A3 gene to influence colorectal cancer risk [126]. This suggests that more fibre seems to be particularly protective in people with certain genotypes. The variant and linked SNPs act as gene expression enhancers. Higher SLC26A3 expression and more fibre allows for stronger protection against CRC. Despite strong epidemiological evidence, fibre intake in many populations remains below recommended levels. The ranges of intake in one study varied from 6.3–21.4 g/day for total dietary fibre, 1.8–15.5 g/day for fruit fibre, 1.9–16.8 g/day for vegetable fibre and 3.0–16.9 g/day for cereal fibre [114]. However, the recommended intake from the National Academy of Medicine is 25–30 g/day of fibre for females and 30–38 g/day for males, indicating a substantial opportunity for CRC risk reduction through dietary improvement [129].

Psyllium (*Plantago ovata*), a gel-forming soluble fibre, exhibits antioxidant, anti-proliferative and anticancer effects in the gut [130]. Psyllium supplementation was found to be more effective in patients with constipation than in healthy controls [131]. The psyllium husk increased the levels of *Lachnospira*, *Faecalibacterium*, *Phascolarctobacterium*, *Veillonella*, and *Sutterella* [131]. These bacteria, specifically *Faecalibacterium* and *Lachnospira*, make butyrate, which has anti-inflammatory effects and can suppress tumour growth [130]. In CRC, these levels are reduced, and increasing them is linked to protection [130,131]. Psyllium fibre also reduces LDL cholesterol (by 0.029 mmol/L for every 1 g/day of psyllium fibre), allowing for less bile acid production [132]. The efficacy of psyllium relies on its viscosity, as polysaccharides solubilize in the small intestine and increase intraluminal viscosity. Therefore, psyllium shortens the time that potential carcinogens and toxic microbial metabolites are in contact with the colonic mucosa.

Resistant starch (RS) is a type of dietary fibre that has some prebiotic properties and can reduce the risk of CRC [133,134]. Resistant starch type 5 (RS5) is part of the resistant starch family; it affects the modulation of the gut microbiota composition. RS can affect the structure of the gut microbiota by fostering the growth of beneficial bacteria populations, elevating the amount of intestinal SCFAs, supporting intestinal barrier restoration, and limiting the translocation of lipopolysaccharides into systemic circulation [133,135]. Propionate, an SCFA, can increase the secretion of glucagon-like peptide-1 from intestinal epithelial cells, thereby enhancing the feeling of satiety. Butyrate, another SCFA, plays a role in chronic inflammation and fortifies the integrity of the intestinal barrier to decrease the chances of CRC. RS can also affect the gut microbiota by reducing the serum levels of branched-chain amino acids (BCAAs) [136]. BCAAs can affect the severity of non-alcoholic fatty liver disease. RS can upregulate the expression of angiopoietin-like protein 4, an inhibitor of pancreatic lipase. This then reduces the absorption of lipids in the intestine. RS5, found naturally in many plant foods, increases the pool of beneficial bacteria like *Clostridia*, *Akkermansia*, *Bifidobacterium*, *Faecalibaculum*, *Prevotella*, *Ruminococcaceae*, and *Roseburia* and reduces harmful bacteria like *Fusobacterium* and *Enterobacter* [134]. A diet rich in RS5 helps balance the gut microbiome and reduce CRC risk.

### 5.5. Probiotics

Probiotics are live microorganisms that can provide a health benefit to the host if consumed in an adequate amount [137]. Probiotics survive the acidity of the stomach and strengthen the gut barrier. Some sources of probiotics include fermented foods like kimchi and supplements like powders. Bacteria found in probiotics, such as *Lactobacillus* and *Bifidobacterium*, inhibit β-glucuronidase, suppress inflammation, modulate apoptosis, and promote IgA production. Probiotic treatment has been shown to reduce CRC-associated bacteria, such as *Prevotella*, *Alloprevotella*, *Fusobacterium*, and *Porphyromonas* [137]. Compared to dairy-based probiotics, plant-based probiotics are typically also rich in fibre, vitamins, and minerals. Probiotics provide health benefits as opposed to other diets that may cause dysbiosis in the gut [137]. This promotes an anti-inflammatory gut environment by modulating gut microbiota and enhancing microbial diversity. The field of plant-based probiotic food is growing, and research on its role in CRC prevention continues.

## 6. Discussion and Future Directions

In this review, we described the most up-to-date evidence on the protective effects of PBDs in CRC development, focusing on the key cellular and molecular mechanisms implicated in colorectal carcinogenesis.

Consumption of red and processed meat has been associated with elevated CRC risk, largely attributed to heme iron, endogenous formation of nitrosamine, and the production of multiple carcinogenic compounds during high-temperature cooking or processing. In contrast, high intake of dietary fibre, abundant in whole grains, legumes, fruits, and vegetables, has been associated with reduced CRC risk through multiple mechanisms. Fibre increases stool bulk and decreases intestinal transit time, thereby reducing mucosal exposure to dietary and microbial carcinogens. Moreover, microbial fermentation of fibre by gut microbiota produces SCFAs, such as butyrate, which exert anti-inflammatory and anti-proliferative effects within the colonic epithelium. Furthermore, plant foods are rich in antioxidants and other phytochemicals that may mitigate oxidative stress and modulate pathways involved in carcinogenesis.

The foregoing evidence suggests that prioritizing PBD patterns represents an evidence-aligned, coherent strategy for promoting both nutritional sufficiency and long-term CRC prevention. More recently, randomized controlled trials, including biomarker-driven interventions, show that a PBD favourably modulates validated CRC risk markers such as inflammation, adiposity, and gut-microbiota-derived micrometabolites, such as SCFAs [39]. Recent large systematic review and meta-analyses of 10 prospective cohort studies with over 1.2 million participants and over 19,000 incident CRC cases provided evidence that adherence to PBD patterns was associated with a significantly lower CRC risk [138]. This protective effect was found to be even stronger when the diet prioritized healthier plant foods, such as legumes, vegetables, fruits, whole grains, and nuts [138]. Other meta-analyses, including over 3.5 million individuals, revealed a 15–18% lower risk of CRC among vegetarians [39,139]. However, these findings are not entirely consistent with prospective cohort analyses reporting no significant association, which may reflect differences in study design, dietary classification, and residual confounding.

Importantly, evidence from large-scale population-based studies indicate that meat consumption is not necessary to maintain adequate nutritional status, and a PBD can meet all established nutritional requirements [33,140]. Clear differentiation between healthy and unhealthy PBDs is also essential to ensure that nutritionally dense whole plant foods are prioritized; however, awareness alone may not translate into behaviour change, as dietary choices are influenced by broader social and environmental factors. Framing dietary recommendations around those minimally processed plant foods supports both adequacy and overall diet quality rather than merely meeting isolated nutrient thresholds. Most PBD patterns are compatible with the principle of nutritional adequacy through dietary diversity. When appropriately planned, they are capable of supplying sufficient macronutrients and essential micronutrients. Such patterns are typically rich in dietary fibre, unsaturated fatty acids, and a wide range of bioactive phytochemicals. Outside of CRC protection, these components have been consistently associated with improved cardiometabolic risk profiles, enhanced insulin sensitivity, and favourable gastrointestinal function [141,142].

To add to the advantages, diets rich in plant foods are associated with lower greenhouse gas emissions, reduced ecological degradation and land use, and decreased water demand compared with a diet that includes animal-sourced products [143,144]. Thus, along with health-promoting value, PBDs also integrate equity and sustainability benefits.

Although several randomized controlled trials have evaluated the effects of plant-based diets on intermediate CRC biomarkers, such as inflammation, insulin resistance, adiposity, and short-chain fatty acid production, there are currently no long-term randomized trials directly comparing PBDs with omnivorous diets for CRC incidence. Existing trials are typically short in duration and focus on mechanistic endpoints rather than cancer outcomes. While the biological plausibility for CRC protection is strong, the absence of long-term randomized evidence remains a significant gap in the literature.

While this review synthesizes a broad range of epidemiological, mechanistic, and clinical evidence, it is important to acknowledge that many of the included studies are observational or mechanistic in nature, and there are relatively few long-term randomized controlled trials that directly compare PBDs with omnivorous diets for CRC outcomes. This limits the ability to draw definitive causal conclusions. A strength of this comprehensive review is the integration of findings across 186 studies, including meta-analyses, large prospective cohorts, and mechanistic research, which together provide a coherent biological rationale for the potential protective effects of PBDs. However, heterogeneity in dietary definitions, reliance on self-reported intake, and variability in study quality represent important limitations.

This existing evidence and emerging findings should be translated into clinical and dietary guidelines to articulate the protective potential of PBDs in reducing CRC risk in a timely and transparent manner. Encouraging increased consumption of minimally processed plant foods provides an inclusive and actionable message, which may foster better adherence to PBD.

Establishing harmonized international surveillance of dietary patterns would facilitate robust meta-analyses and enable the investigation of plant-based dietary effects across diverse populations, geographic regions, and additional cancer types, including stomach, pancreatic, and prostate cancers, for which meat has also been implicated as a probable carcinogen [32]. Thus, the importance of implementing evidence-based PBD patterns has never been more urgent.

## 7. Conclusions

CRC remains a growing global health burden, and dietary modifications represent one of the most evidence-driven, feasible and actionable prevention strategies available. A substantial and converging body of epidemiological, clinical, and mechanistic evidence identifies consuming red and processed meat as a cause of CRC and underscores a PBD as an effective, cost-efficient, and sustainable measure to protect against CRC. A PBD exerts anti-inflammatory, antioxidant, and microbiota-modulating effects that directly influence pathways affecting tumour initiation and progression, such as epithelial proliferation, immune regulation, apoptosis, and genomic stability.

Beyond biological plausibility and epidemiological consistency in CRC risk reduction, a PBD offers affordability and the potential to mitigate health disparities across socio-economic gradients. Integrating plant-forward dietary recommendations into clinical practice, public health strategies, and intersectoral policy initiatives represents a pragmatic and equity-enhancing approach. Collectively, these considerations provide a robust rationale for positioning PBD patterns at the center of contemporary evidence-based nutrition guidance for CRC prevention.

## Data Availability

Data sharing not applicable to this article as no datasets were generated or analysed during the current study.

## Abbreviations

The following abbreviations are used in this manuscript:

AhR	Aryl hydrocarbon receptor
ALA	Alpha-linolenic acid
ATP	Adenosine triphosphate
BCAAs	Branched-chain amino acids
BMI	Body mass index
CI	Confidence interval
CRC	Colorectal cancer
CVD	Cardiovascular disease
DALYs	Disability-adjusted life-years
DHA	Docosahexaenoic acid
EPA	Eicosapentaenoic acid
EPIC	European Prospective Investigation into Cancer and Nutrition
FXR/TGR5	Farnesoid X receptor/G-protein-coupled bile acid receptor 1
HR	Hazard ratio
IARC	International Agency for Research on Cancer
IBD	Inflammatory bowel disease
IGF-1	Insulin-like growth factor 1
IGFBP	Insulin-like growth factor binding protein
IL	Interleukin
JAK/STAT3	Janus kinase/signal transducer and activator of transcription 3
LDL	Low-density lipoprotein
LPSs	Lipopolysaccharides
NF-KB	Nuclear factor kappa B
NO	Nitrogen oxide
NOD1/NOD2	Nucleotide-binding oligomerization domain-containing proteins 1 and 2
NOX	NADPH oxidase
PBD	Plant-based diet
PGEs	Prostaglandins
PPAR-γ	Peroxisome proliferator-activated receptor gamma
ROS	Reactive oxygen species
RS/RS5	Resistant starch/resistant starch type 5
SAT	Subcutaneous adipose tissue
SCFA	Short-chain fatty acid
SIRT1	Sirtuin-1
TLR	Toll-like receptor
TNF-a	Tumour necrosis factor-alpha
TRIP6	Thyroid hormone receptor-interacting protein 6
VAT	Visceral adipose tissue
